# Gemcitabine-Loaded Nanocarrier of Essential Oil from *Pulicaria crispa*: Preparation, Optimization, and In Vitro Evaluation of Anticancer Activity

**DOI:** 10.3390/pharmaceutics14071336

**Published:** 2022-06-24

**Authors:** Sahar M. AlMotwaa, Waad A. Al-Otaibi

**Affiliations:** Department of Chemistry, College of Science and Humanities, Shaqra University, P.O. Box 6974, Al Quwayiyah 19257, Saudi Arabia; w.otaibi@su.edu.sa

**Keywords:** gemcitabine, nanoemulsion, ROS, caspase-3, apoptosis

## Abstract

The limitations of gemcitabine (GEM) in cancer therapy are due to its poor pharmacokinetics, which cause undesired adverse effects. The current study was aimed at investigating the anticancer effect and apoptotic mechanism of synthesized nanoemulsion (NE) containing *Pulicaria crispa* essential oil (PC-EO) and GEM (PC-NE:GEM) on MCF-7 and Hep-G2 cancer cell lines. An optimized NE formulation was selected based on the Box–Behnken method. The droplet size of the optimized PC-NE was 9.93 ± 0.53 nm, but after GEM loading, it was increased to 11.36 ± 0.0.21 nm. Results from FTIR revealed that GEM was successfully loaded onto PC-NE. The antineoplastic effect of PC-NE:GEM on MCF-7 and Hep-G2 cancer cells was increased more than 100-fold relative to that of GEM. A combination index and isobologram based on CompuSyn software revealed the synergistic effect of the formulation produced by a 1:1 ratio combination of PC-NE and GEM. These findings were confirmed by examination of cellular morphologies. The combination formulation strongly induced about 4.48-fold and 2.95-fold increases in apoptosis in MCF-7 and Hep-G2 cells, respectively, when compared with GEM. Moreover, PC-NE:GEM produced a synergistic increase in ROS production in MCF-7 cells (15.23%) and Hep-G2 cells (31.69%), when compared with GEM. In addition, PC-NE:GEM enhanced the activation of the intrinsic apoptosis pathway through upregulation of expressions of p53 and Caspase-3, and downregulation of Bcl-2 expression in MCF-7 cells, while the expressions of Caspase-3, Bax, and p53 were upregulated in HepG2 cells. These results indicate that the GEM-loaded NE containing PC-EO may reduce the dose of GEM and eliminate the associated side effects.

## 1. Introduction

Gemcitabine (GEM) is a chemotherapeutic drug approved by clinicians and used for treating a broad range of cancers, including ovarian, bladder, breast, liver, pancreatic, and nonsmall-cell lung cancers [1]. The anticancer effects of GEM result from the fact that it acts as a deoxycytidine nucleoside analog [1]. It exerts its effect through inhibition of the activity of ribonucleotide reductase, thereby reducing the synthesis of DNA, and preventing the continuation of the cell cycle at the G1/S stage [2]. However, the development of GEM resistance leads to failure of the drug in chemotherapy. The specific reasons behind resistance are unknown. However, several reasons have been advanced, including the promotion of substitutional DNA repair pathways, and deficiencies in drug uptake [3].

Different unfavorable pharmacokinetic parameters may make GEM ineffective. Currently, there is a restriction on clinical trials due to toxicities associated with administered doses, short plasma half-life, and the development of drug resistance [4]. After i.v. administration of GEM, the enzyme deoxycytidine deaminase, which is present in the liver and plasma, metabolizes the drug into inactive 2′, 2′-difluorodeoxyuridine (dFdU). This process presents a short plasma half-life (t½) of 15–20 min [5,6]. Therefore, to order to achieve the required therapeutic concentration, a higher dose of GEM is administered with continuous i.v. infusion. However, clinical trials have demonstrated that high GEM doses are associated with several side effects such as neutropenia, anemia, granulocytopenia, and myelosuppression thrombocytopenia [4,7,8]. The drug GEM is hydrophilic, and it cannot enter the cells through passive diffusion through cell membranes. Thus, for efficient movement across cells, nucleoside transporter systems are needed [4,9].

Nanoemulsions (NEs), nanosized emulsions that are smaller than hundreds of nanometers in size, are applied in different areas such as cosmetics, food, and pharmaceuticals [10,11,12,13]. They have attractive characteristics, including high absorption rates and large surface area per unit volume. The small size of NEs makes them ideal for use as nanomedicines. They can easily penetrate tissues and circulate in the body for a long time, thereby increasing the effectiveness of drugs. Additionally, they are attractive in nanomedicine formulations since they enhance the dissolution of hydrophobic drugs and reduce the severe side effects that may be experienced by patients [11,14].

*Pulicaria crispa* (PC) is a herb traditionally called gethgath. The herb grows annually and perennially in some cases. It belongs to the *Asteraceae* family, and it produces small bright yellow flowers. The plant grows mainly in Asian countries such as Pakistan, India, Saudi Arabia, Iran, Iraq, Kuwait, and Afghanistan. In addition, it is available in a few parts of West and North Africa [15,16]. Egyptians and Saudis use PC for medical purposes as herbal tea, insect repellent, and an anti-inflammatory agent [17]. The plant has essential oils that have valuable roles in foods and medicines. In a previous work, the components and biological activity of the *P. crispa* essential oil were determined. The major compound in the essential oil was identified as β-caryophyllene oxide (33.97%), while the other components were modephene (23.34%), geranyl propionate (6.32%), geranyl isovalerate (6.74%), 4-cadinadiene (5%), humulene (4.05%), and β-caryophyllene (2.73%) [18]. The essential oil (EO) exhibited radical scavenging activity and exerted antibacterial influence on Gram-positive bacteria. Moreover, the essential oil exerted cytotoxic effects on Hep-G2, MCF-7, Coca-2, and HT-29 cell lines, with the most noticeable toxicity in the Hep-G2 cell line [18].

The present study aimed to optimize and characterize NE containing *P. crispa* essential oil (PC-NE), followed by an evaluation of its anticancer and apoptotic effects, together with its ROS scavenging potential. Moreover, the effects of PC-NE on the protein expression levels of Caspase-3, p53, Bax, and Bcl-2 in MCF-7 and Hep-G2 human cancer cell lines were determined, before and after GEM drug loading.

## 2. Materials and Methods

### 2.1. Plant Collection and Essential Oil Extraction

The aerial parts of *Pulicaria crispa* (PC) were collected in Saudi Arabia’s Riyadh region and botanically identified by Essam Al-Sahli Trading Est. for Retail Spices and Herbs. Then, the essential oil extraction process outlined in our earlier work was performed on the PC aerial parts to produce *P. crispa* essential oil [18]. The extracted oil was kept refrigerated at 4 °C before use.

### 2.2. Preparation of PC-NE Formulations

Using a high-pressure homogenization process and various proportions of Tween80 (Tw80) and propylene glycol (PG) (Sigma, St. Louis, MO, USA), *Pulicaria crispa* essential oil (PC-EO), and distilled water, a transparent oil-in-water (O/W) PC-NE formulation was synthesized. In a 5 mL screw cap Pyrex tube, Tw80 and PC-EO were mixed and heated to 60 °C with constant mixing for 5 min until a one-phase emulsion was formed. Then, PG was incorporated into the final mix, followed by 15 min of vortexing at the same speed. The generated transparent/semitransparent solution was then subjected to analysis.

### 2.3. Physical Characterization of NE Formulations

The physical properties of formulated products were determined. A Zetasizer (Malvern Instruments Limited, Malvern, UK) was used to determine the nanodroplet characteristics of PC-NE and PC-NE:GEM. The size of distributed nanodroplets and polydispersity index (PDI) were measured three times. All measurements were taken at room temperature.

### 2.4. Fourier Transform Infrared Spectroscopy (FTIR)

The FTIR analysis was performed to determine the functional groups present in the formulations, together with their interactions. The instrument used for this analysis was the Nicolet iS50 FTIR spectrometer (Thermo Fisher Scientific, Waltham, MA, USA). The GEM, PC-NE, and PC-NE:GEM formulations were analyzed under the 4000–380 cm^−1^ range.

### 2.5. Optimization of PC-NE by Box–Behnken Design

The experimental design and formulation optimization of the PC-NE preparation were performed using the three-level, three-factor Box–Behnken design (Minitab ^®20^ statistical software). This design has been determined to be the best fit for assessing quadratic response surfaces and second-order polynomial models, allowing process optimization with 15 runs. A computer-generated, nonlinear polynomial model quadratic equation that explains the three-factor three-level design is given below:

$$X\;={\;a}_{0}\;+{\;a}_{1}S_{1}\;+{\;a}_{2}S_{2}\;+{\;a}_{3}S_{3}\;+{\;a}_{12}S_{1}S_{2}\;+{\;a}_{13}S_{1}S_{3}\;+{\;a}_{23}S_{2}S_{3}\;+{\;a}_{11}S_{1}^{2\;}+{\;a}_{22}S_{2}^{2}\;+{\;a}_{33}S_{3}^{2}$$

where *X* stands for the dependent variable, a_0_ stands for intercept, a_1_–a_33_ are for regression coefficients determined from individual response data, and S_1_–S_3_ are prefixed independent variable coded levels (S_1_ stands for Tween 80 (%), S_2_ for PC-EO (%), and S_3_ for water (%)). Other elements such as S_1_S_2_, S_1_S_3_, S_2_S_3_, and $S_{i}^{2}$ (i = 1, 2 and 3) represent the interactions of independent variables and quadratic terms. The encoded values and levels of the various independent variables are defined in Table 1.

### 2.6. Preparation of GEM and PC-NE:GEM Formulations

A stock solution of 50 mM GEM (Venus Remedies Limited, Panchkula, India) was prepared by dissolving 13.7 µg of GEM in 1 mL of physiological saline (0.9% (*w*/*v*) NaCl) and 1 mL of the optimized PC-NE, resulting in production of the PC-NE:GEM formulation.

### 2.7. Cell Culture

The hepatocellular carcinoma (Hep-G2) and human breast adenocarcinoma (MCF-7) cell lines were provided by American Type Tissue Culture Collection (ATTCC, Manassas, VA, USA). The cell lines were grown in a 25 cm^2^ cell culture flask with Dulbecco’s modified eagle medium (DMEM) (Gibco Life Technologies, Grand Island, NY, USA), 10% (*v*/*v*) fetal bovine serum (FBS) (Lonza, Walkersville, MD, USA), and 1% (*v*/*v*) penicillin–streptomycin at 37 °C in a 5% CO_2_/95% humidified environment. The medium was changed every 48 h until confluence, and the cells were washed in 2 mL of 10 mM phosphate-buffered saline (PBS), pH 7 (Biodiagnostic, Giza, Egypt). The cells were trypsinized (with 2 mL of trypsin) and incubated at 37 °C.

### 2.8. Cytotoxicity Assay

The cytotoxic effects of GEM, PC-NE, and PC-NE:GEM on MCF-7 and Hep-G2 cells were determined in an assay using 3-(4,5-dimethylthiazol-2-yl)-2,5-diphenyltetrazolium bromide (MTT; Biomatik, ON, Canada). The MCF-7 and Hep-G2 were maintained by incubation in DMEM at 37 °C in a 5% CO_2_ environment. Following incubation, each cell line was seeded at a density of 10^4^ cells per well in a 96-well microplate and cultured for 24 h in the appropriate culture medium. Then, the cells were treated for 24 h with two-fold serial dilutions of the formulations in culture medium. After a 24 h incubation period, 100 μL of MTT reagent (0.5 mg/mL) was added to each well, and incubation was continued for 3 h. Thereafter, the medium was replaced with 100 μL of DMSO to dissolve the resultant formazan crystals, and the absorbance of each well was read at 570 nm in a microplate reader (BioTek Instruments, Bad Friedrichshall, Germany). Wells with cells in culture medium only (untreated cells) served as control, while wells with only media were considered as blank. Experiments were repeated three times for each sample. The % cell viability was calculated in each group (GEM, PC-NE, and PC-NE:GEM) and compared to the % cell viability in control cells.

The fraction affected (Fa) was calculated by dividing the percentage inhibition obtained from the MTT assay by 100. The synergistic, additive, and antagonistic effects of the drugs were determined by subjecting the results to analysis using CompuSyn analysis. The statistical analysis results were provided in terms of combination index (CI) values. A CI value is a numerical representation of the pharmacological interaction between two medications, with CI values more than 1, equal to 1, and less than 1, indicating antagonism, additivity, and synergism, respectively.

### 2.9. Assessment of Cell Morphology

To evaluate the extent of changes in morphology after the MCF-7 and Hep-G2 cells were treated with GEM, PC-NE, and PC-NE:GEM, the MCF-7 and Hep-G2 cells were seeded in a 96-well plate at a density of 10^4^ cells/well for 24 h. Then, after incubation and cell attachment, the cells were treated with 2 doses of each formulation, i.e., GEM (0.3 and 0.5 μM), PC-NE:GEM (0.3% (*v*/*v*) + 0.3 μM and 0.5% (*v*/*v*) + 0.5 μM), and PC-NE (0.3 and 0.5% (*v*/*v*)), for 24 h. Then, the cells were examined under an inverted microscope (Olympus, Southall, UK) at 200× magnification.

### 2.10. Intracellular Determination of Reactive Oxygen Species (ROS)

The intracellular levels of ROS formed were determined using a 2′,7′-dichlorofluorescein diacetate (DCFDA)/H2DCFDA cellular ROS assay kit (ab113851; Abcam, Cambridge, UK). The MCF-7 and Hep-G2 cells were seeded at a density of 10^4^ cells per well, followed by treatment with GEM, PC-NE:GEM, and PC-NE as indicated under 2.9 above. The cells were stained with DCFDA based on the instructions of the kit manual, and fluorescence intensity was measured at 485/535 nm in a multi-microplate reader.

### 2.11. Determination of Apoptosis Using Annexin V-FITC

An Annexin V-FITC Apoptosis Staining/Detection kit (ab14085) (Abcam, UK) was used to measure apoptosis following the manufacturer’s protocol. The MCF-7 and Hep-G2 cells were seeded in 6-well plates at a density of 1 × 10^6^ cells/well, and were treated for 24 h with GEM (0.3 and 0.5 μM), PC-NE:GEM (0.3% (*v*/*v*) + 0.3 μM and 0.5% (*v*/*v*) + 0.5 μM), and PC-NE (0.3 and 0.5% (*v*/*v*)). The cells were collected after centrifugation, resuspended, and mixed with 500 µL of 1X Annexin V binding buffer, followed by sequential staining with 5 µL of Annexin V-FITC and 5 µL propidium iodide. Thereafter, the cells were incubated in the dark for 15 min at room temperature. Then, the cells were analyzed flow cytometrically using an FACScan cytometer (FACSCalibur, Becton Dickinson, San Jose, CA, USA).

### 2.12. Determination Protein Expression Levels of Caspase-3, p53, Bax, and Bcl-2

Each cell line was cultured in triplicate for 24 h and treated with GEM (0.3 and 0.5 μM), PC-NE:GEM (0.3% (*v*/*v*) + 0.3 μM and 0.5% (*v*/*v*) + 0.5 μM), and PC-NE (0.3 and 0.5% (*v*/*v*)). Thereafter, the protein expression levels of Caspase-3, p53, Bax, and Bcl-2 were measured using ELISA assay kits (Abcam, Cambridge, UK) in line with the manufacturer’s protocol.

### 2.13. Statistical Analysis

MINITAB^®^ software (Version 20, Minitab Inc., State College, PA, USA) was used to create the experimental runs in line with the Box–Behnken design of response and surface methodology analysis. Significant effects were determined using MegaStat (version 10.3, Butler University, Indianapolis, IN, USA). Each experiment was carried out in triplicate, and the results are presented as mean ± SEM. Differences were considered statistically significant at *p* ≤ 0.05.

## 3. Results and Discussion

### 3.1. Box–Behnken Model

The Box–Behnken design is a technique dependent on multivariate statistical analysis with a response service model for optimizing the responses with different outcome variables based on two- and three-dimension levels [19]. It is an effective way of minimizing wastage of time and resources in experiments since it combines multiple factors at various levels in a small number of runs [20]. The formation of NEs depends on the dispersion of oil droplets in a continuous aqueous phase using an emulsifying agent [11]. Incorporation of the essential oil has a remarkable effect on droplet size and PDI, thereby affecting the efficacy and stability of the NE formations [21]. The preparation of PC-NE was investigated by conducting fifteen designed experimental batches in line with the Box–Behnken design. The experiments determined the influences of independent factors (i.e., Tween80, PC-EO, and water) on dependent factors (i.e., particle size and PDI). Table 2 shows the details of all experimental batches and software responses.

### 3.2. Box–Behnken Model Analysis

Linear, quadratic, and two-way interaction relationships between the variables were determined with a polynomial regression model, as shown in Table 3. The *p* values for particle size (PS), PDI model, and model terms were significant (*p* < 0.05), indicating the suitability of the model used in performing the experiments. The lack-of-fit test was insignificant for dependent factors (PC and NE; *p* > 0.05). This indicated that the model was well suited. All independent factors did not influence the hydrophilic–lipophilic balance (HLP) of the PC-NEs (data not shown). The HLP ranged between 13.6 and 15.

The *R*^2^ value indicates the degree of fit of the model. When the *R*^2^ value is close to 1, it means that the model has a high fit, with acceptable values close to or greater than 0.8 [22]. In the current study, the values of R^2^ were 0.96 and 0.95 for PS and PDI, respectively. These imply that only 4.39% and 4.54% of the total variables for PS and PDI efficiencies, respectively, were unexplained by the model. The adjusted R^2^ value revealed the excellence of the model. The adjusted R^2^ value represents the degree to which dependent factors are affected by independent factors. A highly adjusted R^2^ value indicates that the independent factors significantly affect dependent factors, and vice-versa. As displayed in Table 3, the values of adjusted R^2^ of droplet size and PDI were 0.88 and 0.87, respectively, indicating the independent factors did not explain only 12.29% and 12.71% of the total variation in the model.

**Figure 1:**
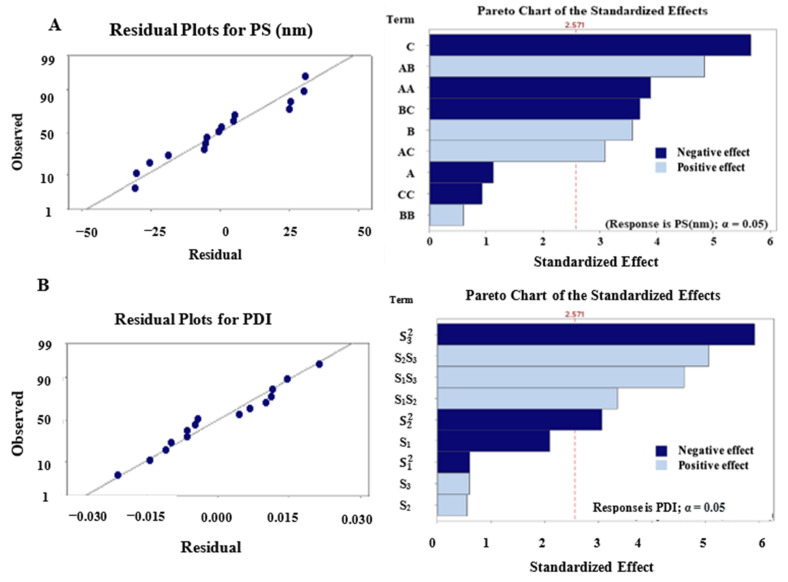
Residual Plots revealing predicted values vs observed values and Pareto charts of effects with independent variables and their relationships. (**A**) PS, (**B**) PDI.

Furthermore, the observed values of R^2^ were comparable to the predicted values of R^2^, as displayed in Figure 1A,B, meaning that the models almost entirely fitted the explanation of the experimental scale investigated. Pareto charts of standardized effects for PS and PDI with independent variables and their relationships are presented in Figure 1A,B. Table 2 shows that the actual droplet sizes of PC-NEs ranged from 9.93 ± 0.33 to 411.90 ± 15.1 nm, while the predicted values ranged from 381.62 to 4.82 nm. The PDI of the prepared PC-NEs was between 0.005 ± 0.0002 and 0.186 ± 0.02, while the predicted value was between 0.027 and 0.18, indicating that the produced NEs were highly dispersed. Values of PDI less than 0.3 are considered indicative of narrow size distribution [23].

The equations produced in the fitted model during optimization using the Minitab software for PS and PDI were:

(1) $$\begin{array}{cc} \mathrm{PS}\;\left(\mathrm{nm}\right)= & 207.8-13.9{\;S}_{1}+44.3{\;S}_{2}-70.1{\;S}_{3}-70.9{\;S}_{1}^{2}+10.9{\;S}_{2}^{2}-16.8{\;S}_{3}^{2}+84.8{\;S}_{1}S_{2}+54.2{\;S}_{1}S_{3}\\ & -64.9{\;S}_{2}S_{3}\; \end{array}$$

(2) $$\begin{array}{cc} \mathrm{PDI}= & 0.174-0.0151{\;S}_{1}+0.004{\;S}_{2}+0.004{\;S}_{3}-0.006{\;S}_{1}^{2}-0.033{\;S}_{2}^{2}-0.063{\;S}_{3}^{2}+0.034{\;S}_{1}S_{2}+0.047{\;S}_{1}S_{3}\\ & +0.052{\;S}_{2}S_{3} \end{array}$$

From Equation (1), it can be predicted that increases in the concentrations of Tw80 and water in the PC-NE formulation had negative effects on the droplet size, as revealed by the positive coefficients S_1_ and S_3_, respectively. Thus, there was a negative correlation between the concentration of PC-EO and droplet size.

From Equation (2), the positive coefficients of S_2_ and S_3_ indicate that increases in PDI were correlated with increases in the concentrations of oil and water, respectively. In contrast, the negative coefficient of S_1_ indicates that decreases in PDI correlated with increases in PC-EO concentration. The changes in droplet size and PDI due to changes in independent factors were described as 2D contour plots and 3D surface plots.

### 3.3. Effect of Interactive Process Parameters

The influence of correlation amongst the three independent factors Tw80, PC-EO, and water on PS and PDI was identified using the response surface method, which was performed with 2D contour plots and 3D response surface plots. The plots were constructed to identify the ideal values of dependent factors within the studied zone based on fixing all factors at a central value, except for two factors.

**Figure 2:**
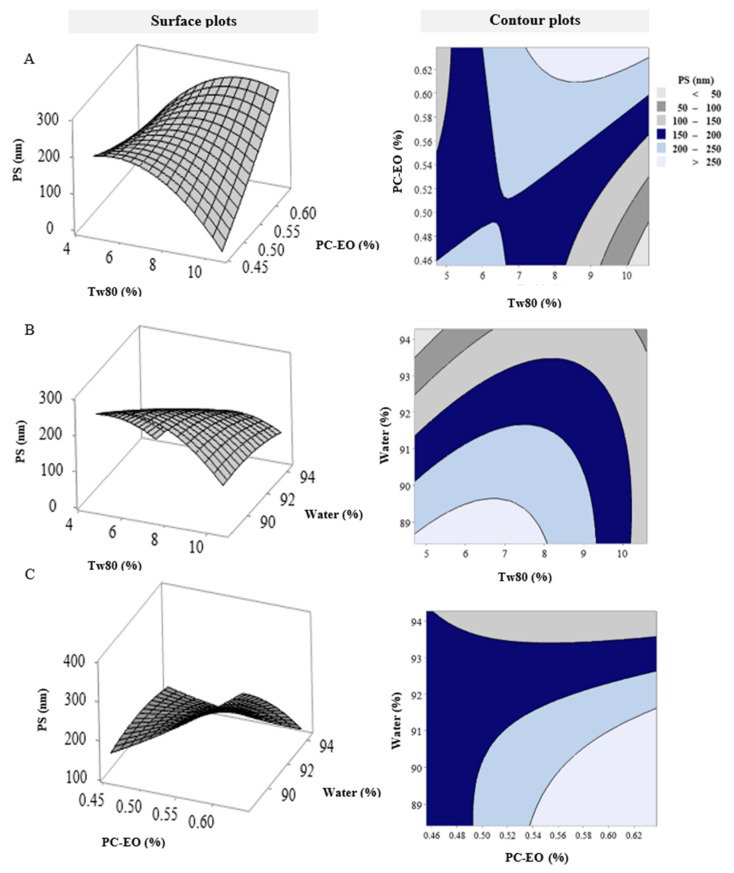
The 3D response surface and contour plots exhibiting combined effects of (**A**) PS versus Tw80 (%) and PC-EO (%), hold value: water (%) 91.34; (**B**) PS versus Tw80 (%) and water (%), hold value: PC-EO (%) 0.55; (**C**) PS versus water (%) and PC-EO (%), hold value: Tw80 (%) 7.66.

The 2D contour plots and 3D surface plots of droplet size in terms of Tw80, PC-EO, and water are presented in Figure 2A. At constant water concentration, an increase in the concentration of Tw80 produced a decrease in droplet size, and an increase in PC-EO concentration had a negative influence on droplet size. The droplet size was inversely proportional to Tw80 concentration, perhaps due to the lowering of interfacial tension between the liquid and oil phases, leading to a decrease in NE droplet size [24]. The increase in the droplet size as a result of increase in PC-EO concentration may be due to a rise in oil concentration and increasing adhesion of NE particles [24]. Figure 2B depicts the effects of Tw80 and water on droplet size at fixed PC-EO concentration. The smallest droplet size was obtained at the lowest concentration of Tw80, which coincided with the highest concentration of water. This influence due to Tw80, which is considered an emulsifying agent; Tw80 has a greater solubility in the water phase than the oil phase [25]. The correlation between the concentration of PC-EO and the amount of water is illustrated in Figure 2C. Increases in the concentration of PC-EO at low levels of water resulted in large increases in droplet size, but decreases in PC-EO concentration, and increases in the amount of water led to sharp declines in droplet size. These relationships due to the rise in oil mass fraction promoted the rate of collisions and coalescence, leading to an increase in the emergence rate of droplets [26]. This effect was reduced as the oil fraction decreased, resulting in decreases in droplet size.

**Figure 3:**
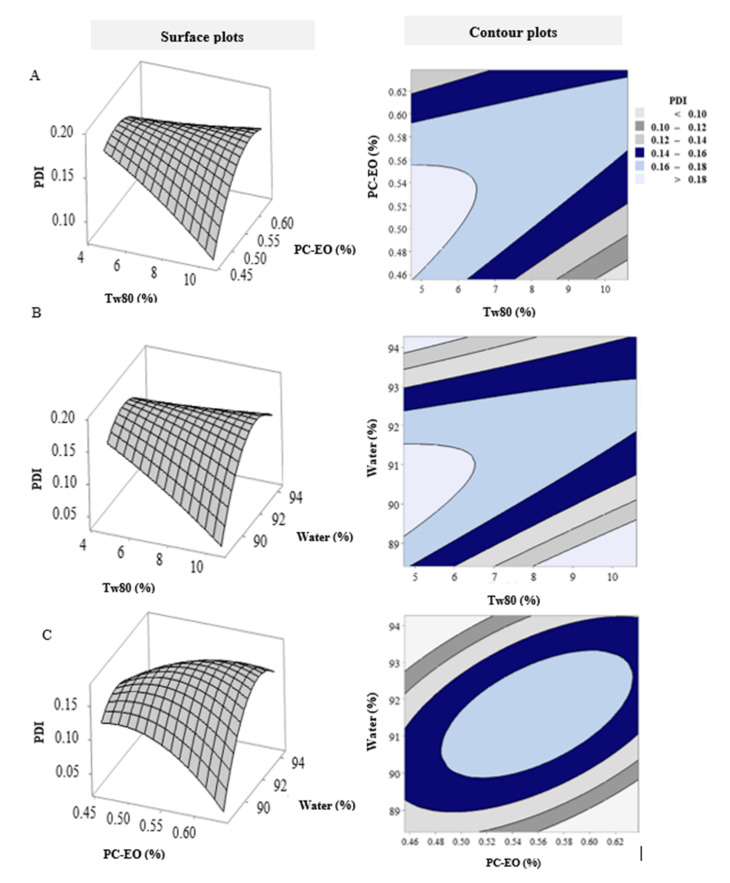
The 3D response surface and contour plots exhibiting combined effects of (**A**) PDI versus Tw80 (%) and PC-EO (%), hold value: water (%) 91.34; (**B**) PDI versus Tw80 (%) and water (%), hold value: PC-EO (%) 0.55; (**C**) PDI versus water (%) and PC-EO (%), hold value: Tw80 (%) 7.66.

The 2D contour plots and 3D response surface plots for PDI are presented in Figure 3. The relationship between Tw80 and PC-EO at fixed water volume is shown in Figure 3A. The highest PDI value was observed at a low concentration of Tw80, which was followed by a sharp pattern of decline as Tw80 concentration increased. On other hand, as the PC-EO concentration decreased from high to low levels, there were gradual decreases in PDI. Figure 3B shows the interaction between Tw80 concentration and water volume at fixed PC-EO concentration. As the Tw80 concentration increased from low to high levels, there were sharp decreases in PDI value. In contrast, as water volume decreased from high to low levels, the PDI value decreased gradually. A previous study established a correlation between increases in Tw80 concentration and low PDI values [27]. This probably implies that an increase in the concentration of the emulsifier resulted in highly homogenous NEs [27,28]. The combined effect of PC-EO and water volume at fixed Tw80 concentration was negative with respect to PC-EO, but it was positive regarding water volume (Figure 3C).

### 3.4. Optimization of PC-NE Formulation

The optimum conditions for PC-NE formulation were selected based on the criteria for attaining minimum values of droplet size and PDI (i.e., less than 0.3). It has been proved that smaller sizes of emulsion droplets may cause high absorption and enhanced bioavailability [29]. The optimum values of selected independent variables obtained using Minitab software were 8.20% Tw80, 0.46% PC-EO, and 91.16% water phase. The observed and predicted values of droplet size for PC-EO (9.93 ± 0.53 and 9.959 nm) and PDI (0.0.068 ± 0.001 and 0.077), respectively, were in good agreement with each other. These results revealed validities of 99.70% and 88.31%, respectively, for the predicted values, thereby proving optimization of the PC-EO formulation.

### 3.5. Development of PC-Loaded NE Formulation

**Figure 4:**
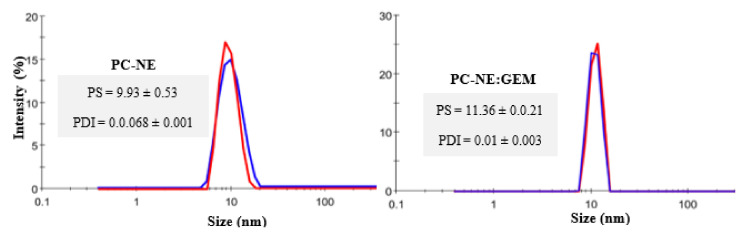
Particles size (nm) and PDI of optimized PC-NE formulation. The values are presented as mean ± SEM (*n* = 3).

The average of droplet size of the PC-NE loaded with GEM (PC-NE:GEM) was 11.36 ± 0.0.21 nm, with a PDI of 0.010 ± 0.003 (Figure 4). The droplet size was increased because of the GEM loading [30,31]. A lower PDI value indicates more uniform droplet size [29].

### 3.6. Determination of PC-NE Interactions Using FTIR

**Figure 5:**
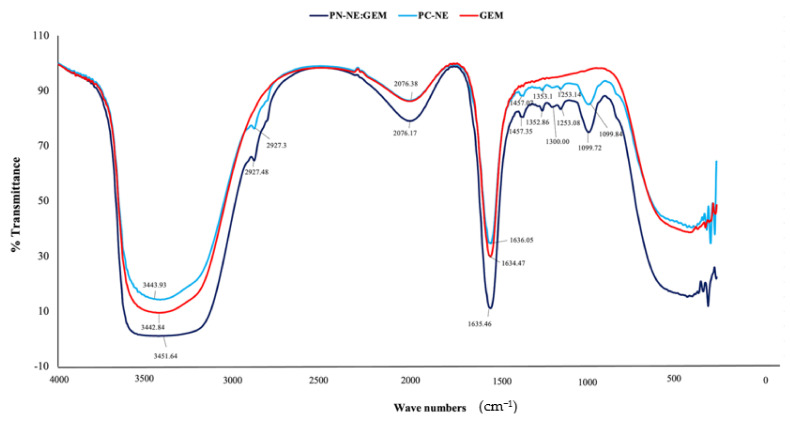
The FTIR spectra of GEM, PC-NE and PC-NE:GEM.

Fourier transform infrared spectroscopy (FTIR) was used to evaluate the interactions between PC-NE and GEM in PC-NE:GEM formulation. All FTIR spectra were analyzed in the range 380–4000 cm^−1^. Variations in the structures of GEM, PC-NE, and PC-NE:GEM are depicted in Figure 5. The most prevalent peaks in GEM, PC-NE, and PC-NE:GEM were observed at 3343, 2076, and 1635 cm^−1^. The 3343 cm^−1^ spectra showed strong and very broad peaks which were attributed to vibration. The stretching of O-H group was strong and very broad at 3343 cm^−1^ [32,33], while C=O stretching was strong and narrowed at 1635 cm^−1^ [34,35]. However, the peak intensities at 3343, 2076, and 1635 cm^−1^ were increased more in PC-NE:GEM than in GEM and PC-NE. Therefore, these increases proved that GEM was successfully loaded onto PC-NE. The other peaks common in PC-NE and PC-NE:GEM observed at 2929, 1460–1353, 1300–1253, and 1099 cm^−1^ were weak, and may include spectral attributes from CH_2_ asymmetric stretching [33,36] and stretching vibrations from C-H [37,38,39], C-O [35,38,40], and C-O-C [35,38,41].

### 3.7. Synergistic Cytotoxicity of PC-NE and GEM in MCF-7 and Hep-G2 Cell Lines

**Figure 6:**
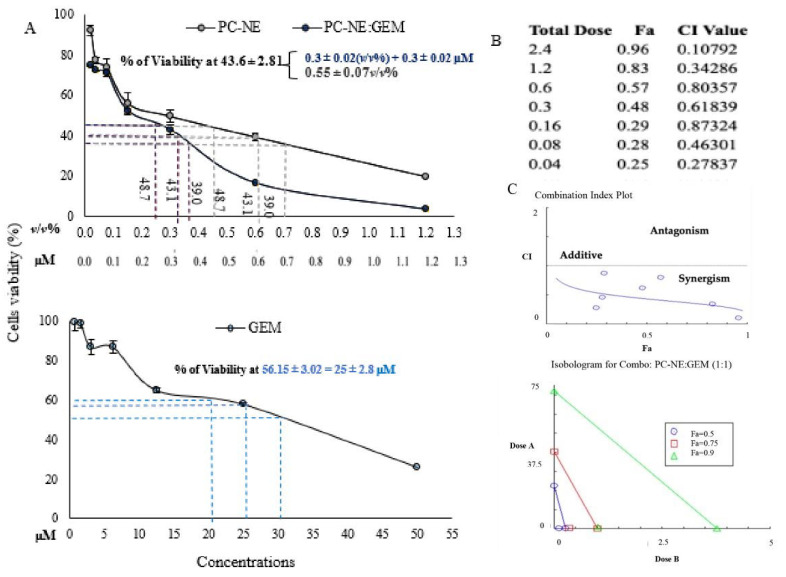

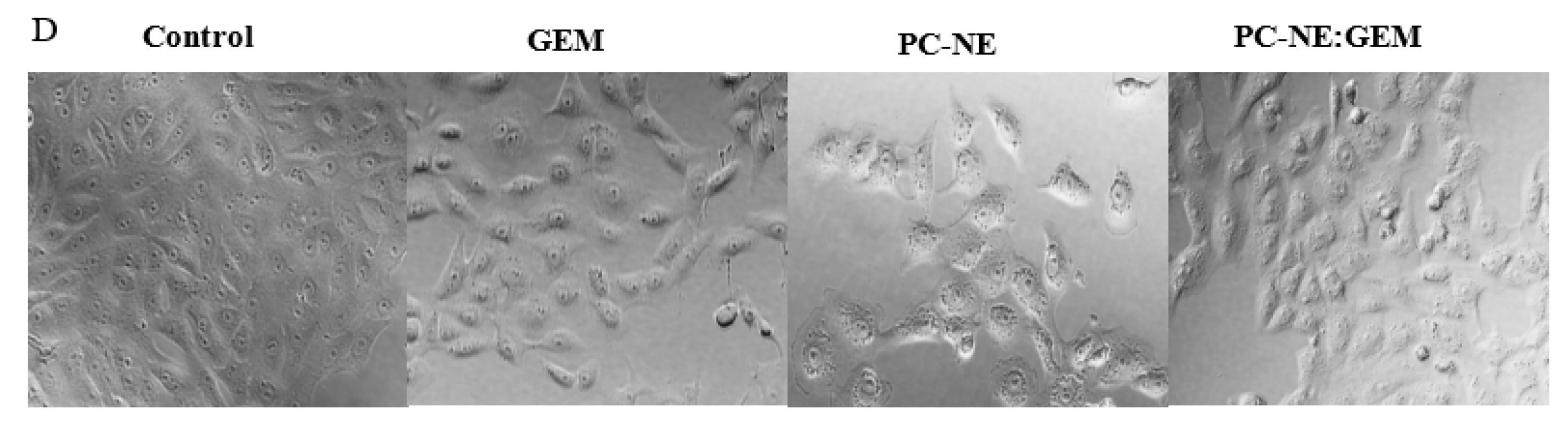
Synergistic effect of PC-NE and GEM on the growth of MCF-7 cells. (**A**) The viability data of MCF-7 cells from the MTT assay are exhibited for PC-NE:GEM, PC-NE, and GEM. Cells were treated with 0–1.2% (*v*/*v*) PC-NE or 0–1.2% (*v*/*v*) + 0–1.2 µM (PC-NE:GEM) or 0–50 µM GEM for 24 h. Data are shown as mean ± SEM (*n* = 3). (**B**) PC-NE synergized with GEM; fraction affected (Fa) and CI values and total doses for PC-NE and GEM combination (constant ratio 1:1) in MCF-7 cells. The CI, isobologram, and Fa values were estimated using CompuSyn software. (**C**) The combination index (CI) plot and isobologram based on the data obtained from the MTT assay. (**D**) Morphological changes in MCF-7 cells in response to GEM, PC-NE, and PC-NE:GEM treatment for 24 h. Cells were treated with the following concentrations: 0.3% (*v*/*v*) PC-NE, 0.3% (*v*/*v*) + 0.3 μM PC-NE(GEM), and 0.3 μM GEM. Magnification: 200×.

**Figure 7:**
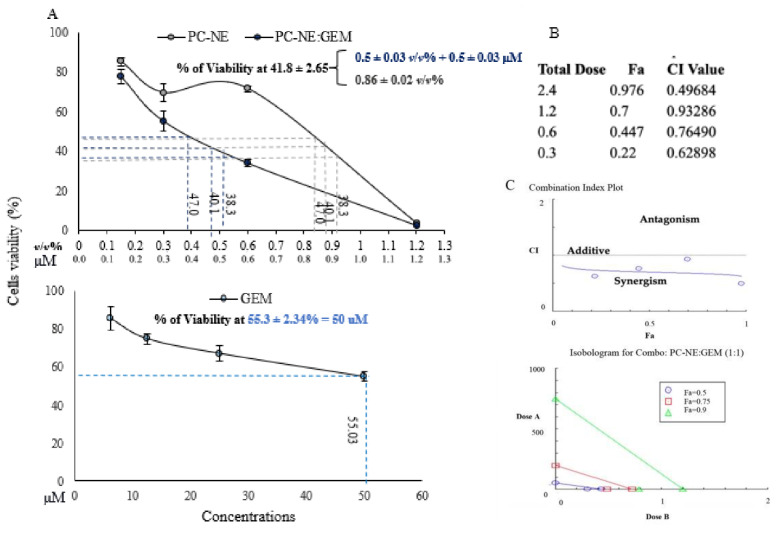

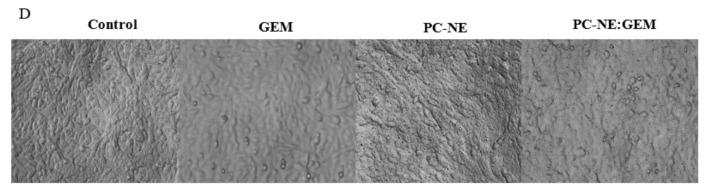
Synergistic effect of PC-NE and GEM on the growth of Hep-G2 cells. (**A**) Effect of PC-NE:GEM, PC-NE, and GEM on the cell viability of Hep-G2 cells, as determined using MTT assay. Cells were treated with 0–1.2% (*v*/*v*) PC-NE or 0–1.2% (*v*/*v*) + 0–1.2 µM (PC-NE:GEM) or 0–50 µM GEM for 24 h. Data are shown as mean ± SEM (*n* = 3). (**B**) Synergy between PC-NE and GEM; fraction affected (Fa), CI value, and total dose of PC-NE and GEM combination (at a constant ratio of 1:1) in Hep-G2 cells. The CI, isobologram, and Fa values were estimated using CompuSyn software. (**C**) The combination index (CI) plot and isobologram were based on the data obtained from the MTT assay. (**D**) Morphological changes in Hep-G2 cells in response to treatments with GEM, PC-NE, and PC-NE:GEM for 24 h. Cells were treated with the following concentrations: 0.5% (*v*/*v*) PC-NE, 0.5% (*v*/*v*) + 0.5 μM (PC-NE:GEM), and 0.5 μM GEM. Magnification: 200×.

To investigate the effects of PC-NE and PC-NE:GEM on the MCF-7 and Hep-G2 cell lines, the cells were treated with varying doses of GEM, PC-NE, and PC-NE:GEM for 24 h. The results are shown in Figure 6A and Figure 7A. Interestingly, the combination of GEM and PC-NE produced more pronounced inhibitory effects on the cell lines than GEM or PC-NE alone.

The IC_56.4_ values of GEM, PC-NE, and PC-NE:GEM for MCF-7 cell line were 38 μM, 0.55 ± 0.07% (*v*/*v*), 0.3 ± 0.02 and (0.3% (*v*/*v*) + 0.3 μM), respectively, while the IC_58.2_ values for the Hep-G2 cell line were <50 μM, 0.86 ± 0.02% (*v*/*v*), and 0.5 ± 0.03 (0.5% (*v*/*v*) + 0.5 μM), respectively. Using IC_56.4_ as a parameter for comparison, the antineoplastic effects of GEM and PC-NE on MCF-7 cells were 127.67 and 0.83 folds lower than that of PC-NE:GEM, respectively. Regarding Hep-G2 cells, PC-NE:GEM exerted a significantly higher cytotoxic effect than GEM and PC-NE. The combination formulation reduced the concentration required to achieve IC58.2 > 100 and 0.72 times, when compared to GEM and PC-NE, respectively.

Furthermore, CompuSyn software was applied to assess the effects of GEM and PC-NE, when used individually and in combination, on the proliferation of MCF-7 and Hep-G2 cells based on the results of MTT assay. The CI values were used to determine synergistic (CI < 1), additive (CI = 1), and antagonistic (CI > 1) effects. The results shown in Figure 6B,C and Figure 7B,C indicate that the CI values ranged from 0.107 to 0.873, and from 0.497 to 0.933 for combination treatments of GEM and PC-NE (1:1 ratio) in MCF-7 and Hep-G2 cells, respectively.

Consequently, the combination of GEM and PC-NE (1:1 ratio) produced a synergistic effect, and all the plotted points (blue circles) are below the black line. Moreover, the isobologram revealed that the combination of GEM and PC-NE (1:1 ratio) produced 90% (green line), 75% (red line), and 50% (blue line) inhibitions. As shown in Figure 6C and Figure 7C, all the combination points within their zone are lower than the additive-effect line on the isobologram in both cell lines, revealing the synergistic effect of PC-NE:GEM.

Therefore, in subsequent investigations, GEM and PC-NE were used at concentrations of 0.5 μM and 0.3% (*v*/*v*), respectively, in MCF-7 cells, and 0.5 μM and 0.5% (*v*/*v*) in Hep-G2 cells, while the concentration of the combined formulation (PC-NE:GEM) was a 1:1 ratio of PC-NE and GEM.

Morphological alterations in the cells due to the various treatments were photographed using an inverted light microscope. Figure 6D and Figure 7D show MCF-7 and Hep-G2 cells after treatment for 24 h. The results in Figure 6D and Figure 7D reveal normal appearance of untreated MCF-7 and Hep-G2 cells. On the other hand, MCF-7 and Hep-G2 cells treated with PC-NE (0.3 and 0.5% (*v*/*v*) and PC-NE:GEM (0.3% (*v*/*v*)+ 0.3μM) and 0.5% (*v*/*v*) + 0.5 μM) resulted in intracellular vacuoles. The MCF-7 cells were more severely affected, as was evident in the fact that the cells became more rounded, with impaired membrane integrity, and loss of communication with neighboring cells. The cells treated with GEM (0.3 μM for MCF-7 cells and 0.5 μM for Hep-G2 cells) exhibited decreases in cell count without appearance of cytoplasmic vacuoles.

The potent anticancer effects of PC-NE and PC-NE:GEM formulations were perhaps due to the capacity of NE to enhance the dispersion of the hydrophobic essential oil in aqueous environments and increase its surface area, which was consistent with the small size of NE [10,11]. Moreover, a previous study in our laboratory showed that PC-EO exerted a strong antitumor effect on various cancer cell lines, including MCF-7, Coca-2, Hep-G2, and HT-29 [18]. The major components (β-caryophyllene oxide and β-caryophyllene) produced potent anticancer effects on various cell lines such as HeLa, HepG2, AGS, SNU-1, A-2780, SNU-16, HT-29, HCT-116, and PANC-1 [42,43]. β-Caryophyllene oxide enhanced ROS generation and activation of different cell signaling pathways, leading to cancer cell death [44]. Other major components, i.e., 4-cadinadiene [45], humulene [46], D-limonene [47], and phytol [48], have been demonstrated to induce Caspase 3 and apoptosis via different signaling pathways.

### 3.8. PC-NE and PC-NE:GEM Induced Apoptosis in MCF-7 and Hep-G2 Cells

**Figure 8:**
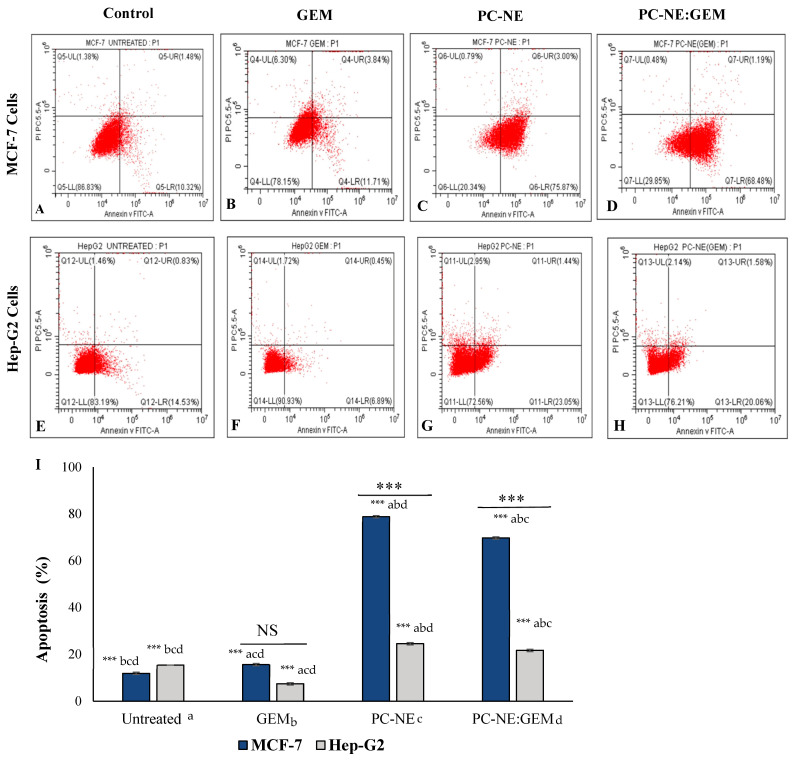
Effect of PC-NE and PC-NE:GEM on apoptosis of MCF-7 and Hep-G2 cells, as was estimated with Annexin V-FITC-PI staining and flow cytometry. (**A**) MCF-7 control cells; (**B**) MCF-7 cells treated with 0.3 μM GEM; (**C**) MCF-7 cells treated with 0.3% *v*/*v* PC-NE; (**D**) MCF-7 cells treated with PC-NE:GEM (0.3% (*v*/*v*) + 0.3 μM); (**E**) Hep-G2 control cells; (**F**) Hep-G2 cells treated with 0.5 μM GEM; (**G**) Hep-G2 cells treated with 0.5% (*v*/*v*) PC-NE; (**H**) Hep-G2 cells treated with PC-NE:GEM (0.5% (*v*/*v*) + 0.5 μM). (**I**) Percentages of apoptosis (early plus late) in MCF-7 and Hep-G2 cells. The statistical differences were determined by independent sample *t*-test, one-way ANOVA, and Tukey’s post hoc test. The data are expressed as mean ± SEM (*n* = 3); *** p  <  0.001; ns = not significant.

The effects of PC-NE and PC-NE:GEM on cell apoptosis were assessed with flow cytometry using annexin-V-FITC staining. As shown in Figure 8, all tested formulations induced apoptosis in MCF-7 and Hep-G2 cells after 24 h treatment with GEM (0.3 μM and 0.5 μM), PC-NE (0.3% (*v*/*v*) and 0.5% (*v*/*v*)), and PC-NE:GEM (0.3% (*v*/*v*) + 0.3 μM, and 0.5% (*v*/*v*) + 0.5 μM). The formulations PC-NE and PC-NE:GEM were more potent in inducing apoptosis in MCF-7 cells than in Hep-G2 cells and when compared with GEM-treated cells (*p* < 0.0001). Overall, PC-NE: GEM triggered 3.34-fold and 2.95-fold increases in apoptosis in MCF-7 and Hep-G2 cell lines, respectively, when compared with GEM, which was consistent with the results of MTT assays and morphological changes of the treated cells. These results indicate that passive targeting using NEs containing PC essential oil as a nanocarrier for GEM improved the drug entry and bioavailability in cancer cells [11].

### 3.9. Estimation of Reactive Oxygen Species (ROS)

**Figure 9:**
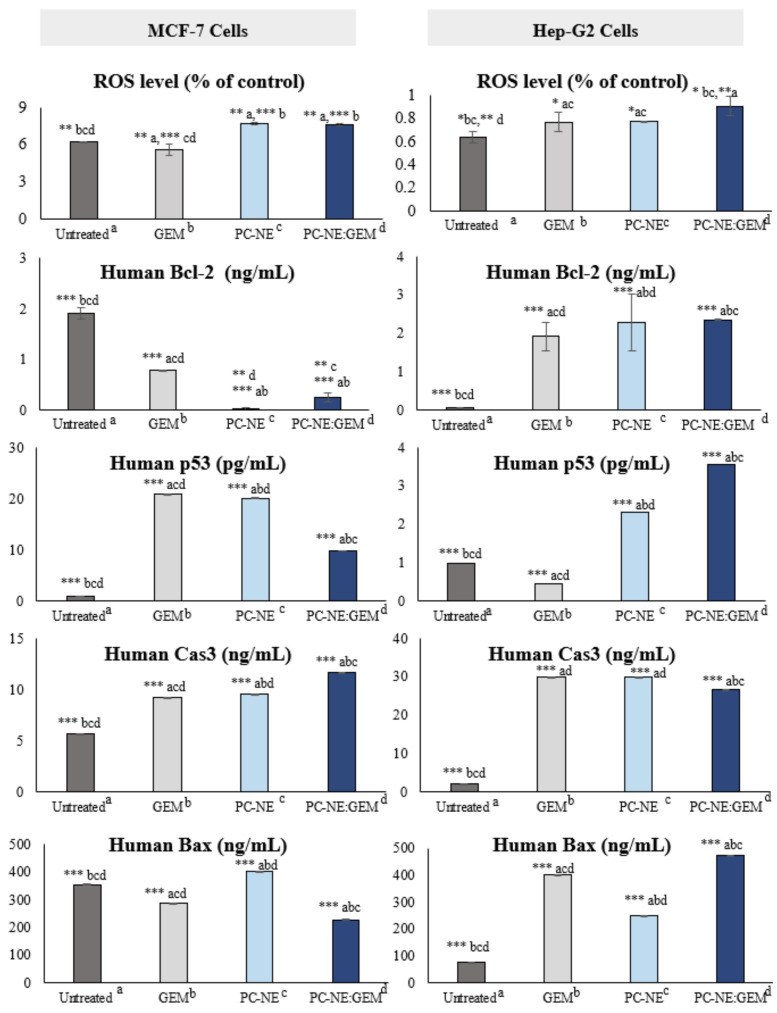
ROS and apoptotic protein levels after treatment with GEM, PC-NE, and PC-NE:GEM for 24 h. Cellular ROS radical detection and ELISA analysis of Caspase-3, p53, Bax, and Bcl-2 protein levels in MCF-7 and Hep-G2 cells. The concentrations of the tested formulations were as follows: 0.3% (*v*/*v*) PC-NE, 0.3% (*v*/*v*) + 0.3 μM of PC-NE:GEM, and 0.3 μM of GEM for MCF-7 cells, and 0.5% (*v*/*v*) of PC-NE, 0.5% (*v*/*v*) + 0.5 μM (PC-NE:GEM) and 0.5 μM GEM (for Hep-G2 cells). Asterisks indicate statistical significance (* *p* < 0.05, ** *p* < 0.01, *** *p* < 0.001). The statistical differences were determined by one-way ANOVA and Tukey’s post hoc test. Data are expressed as mean  ±  SEM (*n* = 3).

The effects of GEM, PC-NE, and PC-NE:GEM at previously indicated concentrations on the potential of MCF-7 and Hep-G2 cells to promote cancer cell death through induction of intracellular ROS were investigated. High cellular ROS impair the functional integrity of cells by interfering with DNA, proteins, and membrane lipids [49], as shown in Figure 9.

PC-NE:GEM produced significant increases in the generation of ROS (approximately 30% and 18% in MCF-7 and Hep-G2 cells, respectively) when compared with untreated cells (*p* < 0.05). When used alone, GEM resulted in significantly reduced ROS production (15.23%) in MCF-7 cells, which was approximately double the decrease in ROS in Hep-G2 cells (31.69%), when compared with PC-NE:GEM; (*p* < 0.05). In the case of PC-NE, the decrease in ROS was significant (15%) in MCF-7 when compared to PC-NE:GEM in MCF-7 (*p* < 0.05), but no significant changes were seen in Hep-G2 cells (*p* > 0.05). These results suggest that combination of the GEM and PC-NE induced a synergistic increase in ROS generation, thereby triggering apoptosis in both cancer cell lines. It has been demonstrated that elevated ROS in cancer cells disrupt DNA (single and double-strand), thereby activating p53 and making cells undergo apoptosis [50].

### 3.10. Effect of PC-NE and PC-NE(GEM) Formulas on Caspase-3, p53, Bax, and Bcl-2 Proteins

The levels of Bcl-2, Bax, p53, and Caspase-3 proteins were quantified in the MCF-7 and Hep-G2 cells with ELSA after treatment with the formulations at the same concentrations as described before. Compared with the untreated group, Figure 9 shows that treatment of MCF-7 cells with GEM, PC-NE, and PC-NE:GEM resulted in significant increases in the expression of p53 (23.27, 22.36, and 10.27 folds; *p* < 0.0001), and Caspase-3 (0.61, 0.67, and 1.07 folds; *p* < 0.0001), respectively. In contrast, significant downregulation of Bcl-2 was observed in cells treated with PC-NE and PC-NE:GEM (76.76 and 6.63 folds, respectively), while GEM alone reduced Bcl-2 by 1.43 folds (*p* < 0.0001). On the other hand, the Bax protein expression was increased 0.13 folds by PC-NE, while GEM and PC-NE:GEM reduced Bax protein expression by 0.19 and 0.36 folds, respectively. In the current study, activation of the apoptosis pathway in MCF-7 cells was concomitant with the downregulation of Bax protein. This indicates that MCF-7 cells required more than 24 h to respond to Bcl-2 signals, and that the promotion of the apoptosis pathway was independent of Bax activation. Stress signals such as ROS production may induce apoptosis by activation of caspase-2, leading to direct liberation of cytochrome c, which activates Apaf-caspase-9, followed by upregulation of Caspase 3 [51]. In addition, the unrepaired DNA damage promoted programmed cell deaths by activation of ATM/ATR and subsequent upregulation of p53, which stimulated caspase-2 [52]. Quantitative apoptotic protein analysis in Hep-G2 cells revealed that PC-NE and PC-NE:GEM markedly activated the expression of Caspase-3 (12.67 and 11.10 folds; *p* < 0.0001), Bax (2.2 and 5.05 folds; *p* < 0.0001), p53 (1.32 and 2.58 folds; *p* < 0.0001), and Bcl-2 (37.93 and 59.20 folds; *p* < 0.0001), respectively, when compared with untreated Hep-G2 cells. These results are shown in Figure 9. The protein levels of Caspase-3, Bax, and Bcl-2 proteins were upregulated by GEM by 12.6, 4.14, and 6.76 folds, respectively, whereas p53 protein was downregulated by 0.54 folds when compared with untreated cells (*p* < 0.0001). It has been reported that among different cancer cell lines, Hep-G2 cells had reduced levels of Bcl-2 after 24 h 3-NC (2-amino-4-(3-nitrophenyl)-3-cyano7-(dimethylamino)-4H-chromene) treatment and almost no Bcl-2 after 48 h [53]. The apoptosis pathway in Hep-G2 cells was induced perhaps by direct activation of Bax protein in response to ROS signal and high expression of p53. The activators of BH3s (BID and BIM) directly interact with the hydrophobic binding groove in Bax-Bak. This changes Bax/Bak conformation and converts homo-dimers to homo-oligomers, which mediates mitochondrial outer member permeabilization (MMOP) and results in the release of cytochrome c and initiation of the caspase cascade [54,55,56]. Collectively, these changes led to upregulation of caspase 3 and apoptosis.

## 4. Conclusions

This investigation demonstrated for the first time that synthesized NE containing PC-EO strongly promoted apoptosis of MCF-7 and Hep-G2 cells. Fifteen NE samples were prepared, out of which an optimized formulation was selected based on the least nanoparticle size and lowest PDI value. Response surface methodology using Box–Behnken design was applied to determine the interactions amongst Tw80, PC-EO, and water, and the effects of these interactions on the average size and PDI of PC-NE. The observed and predicted values of droplet size and PDI for PC-NE were in good agreement with each other. Incorporation of GEM into selected PC-NE formulation significantly reduced GEM dose in both cancer cell lines. The effect was stronger in MCF-7 cell line than in HepG2 cells, as was evident from cytotoxicity assay, morphological changes, and percentage apoptosis. The results revealed that the induction of apoptosis by PC-NE and PC-NE-GEM in MCF-7 cells was due to ROS elevation, upregulations of P53 and caspase 3, and downregulation of Bcl-2. Regarding Hep-G2 cells, the apoptosis was due to upregulations of Bax, p53, and caspase 3 as a result of increased levels of ROS. Reducing the drug dose led to reduction in toxicity and improvement of the therapeutic effects. This effectiveness may be due to the small size and large volume of the surface area of the NEs. Moreover, the NEs enhanced the solubility, absorption, and bioavailability of GEM, which had poor solubility characteristics.

## Figures and Tables

**Table 1:** Factors, symbols, and levels used in the Box–Behnken design for the nanoemulsions involving PC essential oil.

Symbol	Independent Variable	Actual Levels at Coded Factor Levels		
		Low (−1)	Middle (0)	High (1)
S_1_	TW80 (%)	4.71	7.66	10.61
S_2_	PC-EO (%)	0.456	0.547	0.638
S_3_	Water (%)	88.41	91.34	94.26

**Table 2:** BBD parameters with experimental and actual values for the nanoemulsions involving PC essential oil.

Experimental Run	Coded of Independent Factors			Observed Values		Predicted Values	
	(S_1_)	(S_2_)	(S_3_)	PS (nm)	PDI	PS (nm)	PDI
1	−1	−1	0	232.65 ± 11.9	0.169 ± 0.01	202.26	0.180
2	1	−1	0	9.93 ± 0.33	0.067 ± 0.0007	4.82	0.082
3	−1	1	0	115.96 ± 0.93	0.134 ± 0.017	121.08	0.119
4	1	1	0	235.61 ± 4.11	0.168 ± 0.012	263.04	0.158
5	−1	0	−1	235.85 ± 2.43	0.70 ± 0.011	258.27	0.163
6	1	0	−1	121.7 ± 1.6	0.048 ± 0.0002	122.04	0.038
7	−1	0	1	9.95 ± 0.52	0.069 ± 0.0003	9.56	0.077
8	1	0	1	117.16 ± 2.51	0.136 ± 0.02	90.34	0.141
9	0	−1	−1	158.1 ± 2.77	0.126 ± 0.005	162.87	0.122
10	0	1	−1	411.9 ± 15.1	0.005 ± 0.0002	381.16	0.0264
11	0	−1	1	122.6 ± 0.83	0.046 ± 0.0001	152.44	0.027
12	0	1	1	116.1 ± 1.34	0.145 ± 0.023	111.20	0.139
13	0	0	0	189 ± 4.57	0.168 ± 0.005	207.84	0.174
14	0	0	0	201.86 ± 3.75	0.186 ± 0.02	207.84	0.174
15	0	0	0	233.46 ± 5.87	0.166 ± 0.03	207.84	0.174

S_1_ = coded value of Tw80 (%), S_2_ = coded value of PC-EO (%), and S_3_ = coded value of water (%).

**Table 3:** Results of ANOVA for particle sizes (nm) and PDIs of nanoemulsions involving PC essential oil.

		Particles Size (nm)				PDI				
Variable	DF ^1^	Adj SS ^2^	Adj MS ^3^	F-Value	*p*-Value	DF ^1^	Adj SS ^2^	Adj MS ^3^	F-Value	*p*-Value
Model	9	134,095	14,899.5	12.10	0.007 **	9	0.044036	0.004893	11.68	0.007 **
Linear	3	56,521	18,840.4	15.31	0.006 **	3	0.002117	0.000706	1.68	0.284 ^NS^
S_1_	1	1539	1538.7	1.25	0.314 ^NS^	1	0.001837	0.001837	4.39	0.090 ^NS^
S_2_	1	15,673	15,673.2	12.73	0.016 *	1	0.000129	0.000129	0.31	0.604 ^NS^
S_3_	1	39,309	39,309.4	31.93	0.002 **	1	0.000152	0.000152	0.36	0.574 ^NS^
Square	3	20,167	6722.2	5.46	0.049 *	3	0.017554	0.005851	13.97	0.007 **
$S_{1}^{2}$	1	18,585	18,584.6	15.10	0.012 *	1	0.000154	0.000154	0.37	0.570 ^NS^
$S_{2}^{2}$	1	439	439.3	0.36	0.576 ^NS^	1	0.003953	0.003953	9.44	0.028 *
$S_{3}^{2}$	1	1046	1045.8	0.85	0.399 ^NS^	1	0.014668	0.014668	35.02	0.002 **
2-Way Interaction	3	57,407	19,135.8	15.55	0.006 **	3	0.024365	0.008122	19.39	0.003 **
S_1_S_2_	1	28,798	28,797.7	23.39	0.005 **	1	0.004724	0.004724	11.28	0.020 *
S_1_S_3_	1	11,770	11,770.2	9.56	0.027 *	1	0.008882	0.008882	21.20	0.006 **
S_2_S_3_	1	16,839	16,839.4	13.68	0.014 *	1	0.010759	0.010759	25.68	0.004 **
Error	5	6155	1231.0			5	0.002094	0.000419		
Lack-of-Fit	3	5149	1716.2	3.41	0.235	3	0.001901	0.000634	6.55	0.135
Pure Error	2	1006	503.1			2	0.000193	0.000097		
Total	14	140,250				14	0.046130			
R^2^	95.61%					95.46%				
Adjusted R^2^	87.71%					87.29%				

S_1_ = coded value of Tw80 (%), S_2_ = coded value of PC-EO (%), and S_3_ = coded value of water (%). ^1^ DF—degrees of freedom. ^2^ Adj SS: adjusted sum of square. ^3^ Adj MS: adjusted mean square; * *p* > 0.05, ** *p* > 0.01, and NS: nonsignificant *p* < 0.05.

## Data Availability

Not applicable.

## References

[1] Pandit B., Royzen M. (2022). Recent Development of Prodrugs of Gemcitabine. Genes.

[2] Huang P., Chubb S., Hertel L.W., Grindey G.B., Plunkett W. (1991). Action of 2′, 2′-difluorodeoxycytidine on DNA synthesis. Cancer Res..

[3] Adamska A., Elaskalani O., Emmanouilidi A., Kim M., Razak N.B.A., Metharom P., Falasca M. (2018). Molecular and cellular mechanisms of chemoresistance in pancreatic cancer. Adv. Biol. Regul..

[4] Paroha S., Verma J., Dubey R.D., Dewangan R.P., Molugulu N., Bapat R.A., Sahoo P.K., Kesharwani P. (2021). Recent advances and prospects in gemcitabine drug delivery systems. Int. J. Pharm..

[5] Moog R., Burger A., Brandl M., Schüler J., Schubert R., Unger C., Fiebig H., Massing U. (2002). Change in pharmacokinetic and pharmacodynamic behavior of gemcitabine in human tumor xenografts upon entrapment in vesicular phospholipid gels. Cancer Chemother. Pharmacol..

[6] Reid J.M., Qu W., Safgren S.L., Ames M.M., Krailo M.D., Seibel N.L., Kuttesch J., Holcenberg J. (2004). Phase I trial and pharmacokinetics of gemcitabine in children with advanced solid tumors. J. Clin. Oncol..

[7] Dasanu C.A. (2008). Gemcitabine: Vascular toxicity and prothrombotic potential. Expert Opin. Drug Saf..

[8] Boven E., Schipper H., Erkelens C., Hatty S.A., Pinedo H.M. (1993). The influence of the schedule and the dose of gemcitabine on the anti-tumour efficacy in experimental human cancer. Br. J. Cancer.

[9] Hammond J.R., Lee S., Ferguson P.J. (1999). [3H] gemcitabine uptake by nucleoside transporters in a human head and neck squamous carcinoma cell line. J. Pharmacol. Exp. Ther..

[10] Harwansh R.K., Deshmukh R., Rahman M.A. (2019). Nanoemulsion: Promising nanocarrier system for delivery of herbal bioactives. J. Drug Deliv. Sci. Technol..

[11] Wilson R.J., Li Y., Yang G., Zhao C. (2021). Nanoemulsions for drug delivery. Particuology.

[12] AlMotwaa S.M., Alkhatib M.H., Alkreathy H.M. (2020). Incorporating ifosfamide into salvia oil-based nanoemulsion diminishes its nephrotoxicity in mice inoculated with tumor. Bioimpacts.

[13] Al-Otaibi W.A., Alkhatib M.H., Wali A.N. (2020). Protective role of nanoemulsion containing roman chamomile oil against mitomycin C-induced toxicity in Ehrlich ascites carcinoma bearing mice. Indian J. Biochem. Biophys..

[14] Sutradhar K.B., Amin M.L. (2013). Nanoemulsions: Increasing possibilities in drug delivery. Eur. J. Nanomed..

[15] Al-Rawi A. (1987). Flora of Kuwait.

[16] Boulos L. (2002). Flora of Egypt.

[17] Ross S.A., El Sayed K.A., El Sohly M.A., Hamann M.T., Abdel-Halim O.B., Ahmed A.F., Ahmed M.M. (1997). Phytochemical analysis of Geigeria alata and Francoeuria crispa essential oils. Planta. Med..

[18] AlMotwaa S.M., Al-Otaibi W.A. (2022). Determination of the chemical composition and antioxidant, anticancer, and antibacterial properties of essential oil of Pulicaria crispa from Saudi Arabia. J. Indian Chem. Soc..

[19] Ferreira S.C., Bruns R.E., Ferreira H.S., Matos G.D., David J.M., Brandão G.C., da Silva E.P., Portugal L.A., Dos Reis P.S., Souza A.S. (2007). Box-Behnken design: An alternative for the optimization of analytical methods. Anal. Chim. Acta..

[20] Kaiser S., Verza S.G., Moraes R.C., Pittol V., Peñaloza E.M.C., Pavei C., Ortega G.G. (2013). Extraction optimization of polyphenols, oxindole alkaloids and quinovic acid glycosides from cat’s claw bark by Box–Behnken design. Ind. Crops Prod..

[21] Choudhury H., Gorain B., Chatterjee B., K Mandal U., Sengupta P., K Tekade R. (2017). Pharmacokinetic and pharmacodynamic features of nanoemulsion following oral, intravenous, topical and nasal route. Curr. Pharm. Des..

[22] Dudhipala N., Veerabrahma K. (2015). Pharmacokinetic and pharmacodynamic studies of nisoldipine-loaded solid lipid nanoparticles developed by central composite design. Drug Dev. Ind. Pharm..

[23] Golfomitsou I., Mitsou E., Xenakis A., Papadimitriou V. (2018). Development of food grade O/W nanoemulsions as carriers of vitamin D for the fortification of emulsion based food matrices: A structural and activity study. J. Mol. Liq..

[24] Kapoor H., Aqil M., Imam S.S., Sultana Y., Ali A. (2019). Formulation of amlodipine nano lipid carrier: Formulation design, physicochemical and transdermal absorption investigation. J. Drug Deliv. Sci. Technol..

[25] Prieto C., Calvo L. (2013). Performance of the biocompatible surfactant Tween 80, for the formation of microemulsions suitable for new pharmaceutical processing. J. Appl. Chem..

[26] Rayner M., Dejmek P. (2015). Engineering Aspects of Food Emulsification and Homogenization.

[27] Pongsumpun P., Iwamoto S., Siripatrawan U. (2020). Response surface methodology for optimization of cinnamon essential oil nanoemulsion with improved stability and antifungal activity. Ultrason. Sonochem..

[28] Cheong A.M., Tan C.P., Nyam K.L. (2018). Emulsifying conditions and processing parameters optimisation of kenaf seed oil-in-water nanoemulsions stabilised by ternary emulsifier mixtures. Food Sci. Technol. Int..

[29] McClements D.J. (2021). Advances in edible nanoemulsions: Digestion, bioavailability, and potential toxicity. Prog. Lipid Res..

[30] Al-otaibi W. (2021). Rosemary oil nano-emulsion potentiates the apoptotic effect of mitomycin C on cancer cells in vitro. Pharmacia.

[31] AlMotwaa S.M. (2021). Coupling Ifosfamide to nanoemulsion-based clove oil enhances its toxicity on malignant breast cancer and cervical cancer cells. Pharmacia.

[32] Moghaddasi F., Housaindokht M.R., Darroudi M., Bozorgmehr M.R., Sadeghi A. (2018). Synthesis of nano curcumin using black pepper oil by O/W Nanoemulsion Technique and investigation of their biological activities. Lwt.

[33] Yilmaz M.T., Yilmaz A., Akman P.K., Bozkurt F., Dertli E., Basahel A., Al-Sasi B., Taylan O., Sagdic O. (2019). Electrospraying method for fabrication of essential oil loaded-chitosan nanoparticle delivery systems characterized by molecular, thermal, morphological and antifungal properties. Innov. Food Sci. Emerg. Technol..

[34] Yasmeen S., Kabiraz M., Saha B., Qadir M., Gafur M., Masum S. (2016). Chromium (VI) ions removal from tannery effluent using chitosan-microcrystalline cellulose composite as adsorbent. Int. Res. J. Pure Appl. Chem..

[35] Yang J., Lin J., Yu Q., Zhong H., Liu Y., Chen L. (2018). Study on Adsorption of Cu2 in WasteWater by Residual Sludge as Adsorbent. IOP Conf. Ser. Mater. Sci. Eng..

[36] Ravindran B., Sravani R., Mandal A.B., Contreras-Ramos S.M., Sekaran G. (2013). Instrumental evidence for biodegradation of tannery waste during vermicomposting process using Eudrilus eugeniae. J. Therm. Anal. Calorim..

[37] Panwar K., Jassal M., Agrawal A.K. (2015). In situ synthesis of Ag–SiO2 Janus particles with epoxy functionality for textile applications. Particuology.

[38] Choowang R., Lin J., Zhao G.J. (2019). Composition of Water-insoluble Extract from Oil Palm Trunk Liquefaction Using Polyhydric Alcohol. BioResources.

[39] Farber C., Li J., Hager E., Chemelewski R., Mullet J., Rogachev A.Y., Kurouski D. (2019). Complementarity of raman and infrared spectroscopy for structural characterization of plant epicuticular waxes. ACS Omega.

[40] Vahur S., Kriiska A., Leito I. (2011). Investigation of the adhesive residue on the flint insert and the adhesive lump found from the Pulli Early Mesolithic settlement site (Estonia) by micro-ATR-FT-IR spectroscopy. Est. J. Archaeol..

[41] Ibrahim M., Osman O., Mahmoud A.A. (2011). Spectroscopic analyses of cellulose and chitosan: FTIR and modeling approach. J. Comput. Theor. Nanosci..

[42] Jun N.J., Mosaddik A., Moon J.Y., Ki-Chang J., Dong-Sun L., Ahn K.S., Cho S.K. (2011). Cytotoxic activity of [beta]-Caryophyllene oxide isolated from jeju guava (Psidium cattleianum sabine) leaf. Rec. Nat. Prod..

[43] Dahham S.S., Tabana Y.M., Iqbal M.A., Ahamed M.B., Ezzat M.O., Majid A.S., Majid A.M. (2015). The anticancer, antioxidant and antimicrobial properties of the sesquiterpene β-caryophyllene from the essential oil of Aquilaria crassna. Molecules.

[44] LoPiccolo J., Blumenthal G.M., Bernstein W.B., Dennis P.A. (2008). Targeting the PI3K/Akt/mTOR pathway: Effective combinations and clinical considerations. Drug Resist. Updat..

[45] Hui L., Zhao G., Zhao J. (2015). δ-Cadinene inhibits the growth of ovarian cancer cells via caspase-dependent apoptosis and cell cycle arrest. Int. J. Clin. Exp. Pathol..

[46] Chen H., Yuan J., Hao J., Wen Y., Lv Y., Chen L., Yang X. (2019). α-Humulene inhibits hepatocellular carcinoma cell proliferation and induces apoptosis through the inhibition of Akt signaling. Food Chem. Toxicol..

[47] Anandakumar P., Kamaraj S., Vanitha M.K. (2021). D-limonene: A multifunctional compound with potent therapeutic effects. J. Food Biochem..

[48] Islam M.T., Ali E.S., Uddin S.J., Shaw S., Islam M.A., Ahmed M.I., Shill M.C., Karmakar U.K., Yarla N.S., Khan I.N. (2018). Phytol: A review of biomedical activities. Food Chem. Toxicol..

[49] Aggarwal V., Tuli H.S., Varol A., Thakral F., Yerer M.B., Sak K., Varol M., Jain A., Khan M., Sethi G. (2019). Role of reactive oxygen species in cancer progression: Molecular mechanisms and recent advancements. Biomolecules.

[50] Shi T., Dansen T.B. (2020). Reactive oxygen species induced p53 activation: DNA damage, redox signaling, or both?. Antioxid. Redox Signal..

[51] Guo Y., Srinivasula S.M., Druilhe A., Fernandes-Alnemri T., Alnemri E.S. (2002). Caspase-2 induces apoptosis by releasing proapoptotic proteins from mitochondria. J. Biol. Chem..

[52] Vigneswara V., Ahmed Z. (2020). The role of caspase-2 in regulating cell fate. Cells.

[53] Naseri M.H., Mahdavi M., Davoodi J., Tackallou S.H., Goudarzvand M., Neishabouri S.H. (2015). Up regulation of Bax and down regulation of Bcl2 during 3-NC mediated apoptosis in human cancer cells. Cancer Cell Int..

[54] Chen H., Kanai M., Inoue-Yamauchi A., Tu H., Huang Y., Ren D., Kim H., Takeda S., Reyna D.E., Chan P.M. (2015). An interconnected hierarchical model of cell death regulation by the BCL-2 family. Nat. Cell Biol..

[55] Jeng P.S., Inoue-Yamauchi A., Hsieh J.J., Cheng E.H. (2018). BH3-dependent and independent activation of BAX and BAK in mitochondrial apoptosis. Curr. Opin. Physiol..

[56] Singh R., Letai A., Sarosiek K. (2019). Regulation of apoptosis in health and disease: The balancing act of BCL-2 family proteins. Nat. Rev. Mol. Cell Biol..

