# Correction: AlMotwaa, S.M.; Al-Otaibi, W.A. Gemcitabine-Loaded Nanocarrier of Essential Oil from *Pulicaria crispa*: Preparation, Optimization, and In Vitro Evaluation of Anticancer Activity. *Pharmaceutics* 2022, *14*, 1336

**DOI:** 10.3390/pharmaceutics17050646

**Published:** 2025-05-14

**Authors:** Sahar M. AlMotwaa, Waad A. Al-Otaibi

**Affiliations:** Department of Chemistry, College of Science and Humanities, Shaqra University, P.O. Box 6974, Al Quwayiyah 19257, Saudi Arabia; w.otaibi@su.edu.sa

**Error in Figure**

In the original publication [1], there was a mistake in Figure 8 as published. Figure 8G–H was duplicated incorrectly in the proof version. The corrected Figure 8H appears below. The authors state that the scientific conclusions are unaffected. This correction was approved by the Academic Editor. The original publication has also been updated.

## Figures and Tables

**Figure 8:**
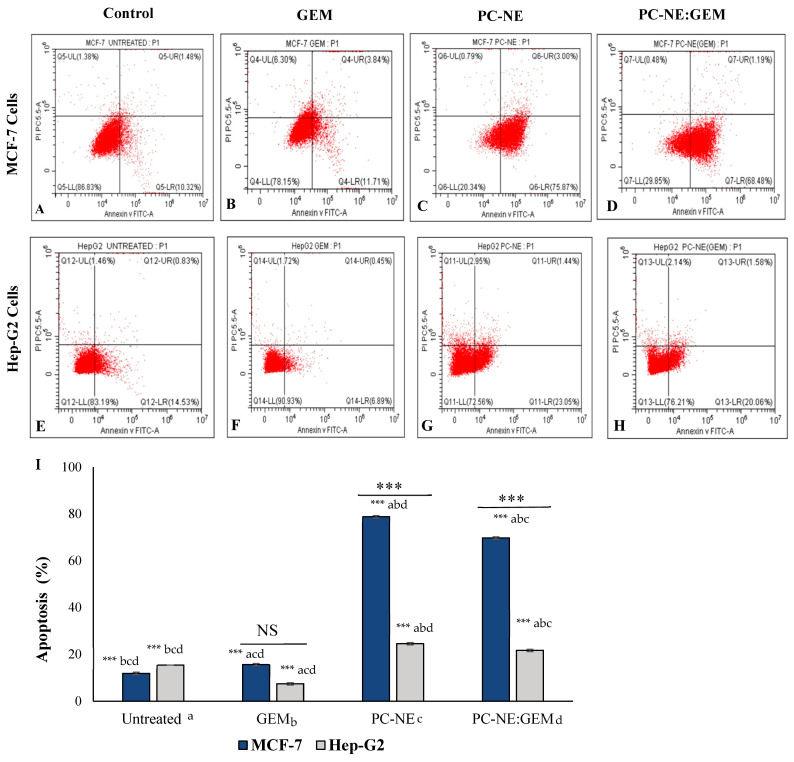
Effect of PC-NE and PC-NE:GEM on apoptosis of MCF-7 and Hep-G2 cells, as was estimated with Annexin V-FITC-PI staining and flow cytometry. (**A**) MCF-7 control cells; (**B**) MCF-7 cells treated with 0.3 μM GEM; (**C**) MCF-7 cells treated with 0.3% *v*/*v* PC-NE; (**D**) MCF-7 cells treated with PC-NE:GEM (0.3% (*v*/*v*) + 0.3 μM); (**E**) Hep-G2 control cells; (**F**) Hep-G2 cells treated with 0.5 μM GEM; (**G**) Hep-G2 cells treated with 0.5% (*v*/*v*) PC-NE; (**H**) Hep-G2 cells treated with PC-NE:GEM (0.5% (*v*/*v*) + 0.5 μM). (**I**) Percentages of apoptosis (early plus late) in MCF-7 and Hep-G2 cells. The statistical differences were determined by independent sample *t*-test, one-way ANOVA, and Tukey’s post hoc test. The data are expressed as mean ± SEM (*n* = 3); *** *p*  <  0.001; ns = not significant.
